# Synthesis, Characterization, and Modeling of Aligned ZnO Nanowire-Enhanced Carbon-Fiber-Reinforced Composites

**DOI:** 10.3390/ma15072618

**Published:** 2022-04-02

**Authors:** Jingyu Wang, Parisa Marashizadeh, Binbin Weng, Preston Larson, M. Cengiz Altan, Yingtao Liu

**Affiliations:** 1 School of Aerospace & Mechanical Engineering, University of Oklahoma, Norman, OK 73019, USA; jingyuwang@ou.edu (J.W.); parisa.m@ou.edu (P.M.); altan@ou.edu (M.C.A.); 2 School of Electrical and Computer Engineering, University of Oklahoma, Norman, OK 73019, USA; binbinweng@ou.edu; 3 Samuel Roberts Noble Microscopy Laboratory, University of Oklahoma, Norman, OK 73019, USA; plarson@ou.edu

**Keywords:** nanoparticles, carbon fiber, interfacial properties, multiscale modeling, hybrid composite

## Abstract

This paper presents the synthesis, characterization, and multiscale modeling of hybrid composites with enhanced interfacial properties consisting of aligned zinc oxide (ZnO) nanowires and continuous carbon fibers. The atomic layer deposition method was employed to uniformly synthesize nanoscale ZnO seeds on carbon fibers. Vertically aligned ZnO nanowires were grown from the deposited nanoscale seeds using the low-temperature hydrothermal method. Morphology and chemical compositions of ZnO nanowires were characterized to evaluate the quality of synthesized ZnO nanowires in hybrid fiber-reinforced composites. Single fiber fragmentation tests reveal that the interfacial shear strength (IFSS) in epoxy composites improved by 286%. Additionally, a multiscale modeling framework was developed to investigate the IFSS of hybrid composites with radially aligned ZnO nanowires. The cohesive zone model (CZM) was implemented to model the interface between fiber and matrix. The damage behavior of fiber was simulated using the ABAQUS user subroutine to define a material’s mechanical behavior (UMAT). Both experimental and analytical results indicate that the hierarchical carbon fibers enhanced by aligned ZnO nanowires are effective in improving the key mechanical properties of hybrid fiber-reinforced composites.

## 1. Introduction

Advances in reinforcements fibers and polymers have led to the development of high-performance structural composites [1]. Multiple types of fibers have been applied as reinforcing materials, such as carbon fiber, glass fiber, and aramid fiber. Since the mechanical properties of composites depend on the constituent materials as well as on the interfacial properties between fiber and polymer matrix, the research of advanced interfaces and load transfer mechanisms in composites has received considerable attention. Multiple surface treatment methods have been investigated to improve the bonding at the interface of fiber and matrix in carbon-fiber-reinforced polymer (CFRP) composites. In general, the interfacial properties of CFRP composites are improved by following three strategies: (1) to enhance the chemical interactions at the interface of fiber and matrix; (2) to increase load transfer area on fibers; (3) to simultaneously integrate chemical interactions and increased contact area of structural fibers. For example, chemical oxidation can remove weak outer layers of fibers and provide functional groups to generate strong chemical bonds between fiber and matrix using chemical interactions [2,3]. Nonoxidative treatment methods, such as grafting, involve the deposition of new materials on fibers, resulting in a thin layer of coating on fibers to improve load transfer capability [4]. Additionally, the high-energy irradiation method has been used to cure polymer matrix in composites, creating oxygen functional groups on carbon fibers and tailoring surface roughness for increased contact area between fiber and matrix [5]. However, the surface oxidation, plasma treatment, and other chemical processing of carbon fibers can influence the graphite structures in carbon fibers and weaken the overall composite properties.

Recently, whiskerization has been a popular approach to enhance the strength of structural fibers with the high strength of nanostructures [6]. Various nanoparticles, including aligned carbon nanotubes (CNTs), silicon carbide (SiC) nanowires, zinc oxide (ZnO) nanowires, have been synthesized on carbon fibers as interphase for the enhancement of interfacial properties [7,8,9]. The employment of CNTs and SiC nanowires on continuous structural fibers increases their surface area, which can lead to improved mechanical interlocking and load transfer capabilities between fiber and matrix in composites. However, these approaches are challenged by the potential degradation of carbon fibers caused by the harsh nanoparticle synthesis procedures. The growth of CNTs and SiC nanowires using chemical vapor deposition (CVD) methods are usually carried out above 900 °C. Certain high processing temperatures can cause significant fiber degradation, leading to the decrease in in-plane mechanical properties of CFRP composites [10]. In addition, the catalyst used during the synthesis of CNTS can also significantly reduce the tensile strength of carbon fibers [11].

To overcome these problems, Lin et al. developed a two-step hydrothermal method at the low temperature of 90 °C to vertically grow ZnO nanowires on carbon fiber [12]. It was demonstrated that the interfacial shear strength (IFSS) was improved by 110% when ZnO nanowires worked as interphase, without degradation of the tensile strength of carbon fibers. Additionally, Ehlert et al. synthesized ZnO nanowires as interphase to carbon fibers and explained the chemical mechanism of adhesion [9]. Both experiment and molecule dynamics simulations were employed to prove that the ketone groups improved the adhesion between ZnO and graphite [13,14]. The atomistic traction–separation analysis indicated enhanced bonding between fiber and matrix incorporating ZnO nanowires [15]. Moreover, Patterson et al. directly measured the adhesive force between ZnO nanoparticles and highly oriented pyrolytic graphite (HOPG) using atomic force microscopy (AFM) to validate that ZnO was a strong interface in hybrid composites [16]. Marashizadeh et al. developed a multiscale analysis framework to simulate the interaction of ZnO nanowires between carbon fibers and matrix [17] and between CFRP laminates [18]. They confirmed that the existence of ZnO nanowires enhanced the load transferring capabilities between carbon fibers and the matrix.

Using the two-step hydrothermal method, the mechanical properties of carbon fibers were preserved due to the low-temperature aqueous growth, compared with the traditional CVD growth of CNTs [12]. In the first step, the seed layer growth of ZnO nanoparticles is the most important to determine the second step of ZnO nanowires growth [19]. The non-uniform ZnO seed layer will lead to the large size of ZnO nanowires and affect the morphologies of ZnO nanowires. For the first step, a dipping method was used to grow the ZnO nanoparticles, which has a limitation of large grain size of ZnO nanoparticles and difficult uniformity. Compared with dipping, atomic layer deposition (ALD) is promising for the growth of ZnO nanoparticles due to its excellent uniformity, high degree of conformity, atomic-scale thickness controllability, perfect stoichiometric uniformity, low impurity contamination, and low growth temperature, which can be as low as 100 °C, close to the dipping method [20,21]. ZnO nanowires synthesized on carbon fabric using ALD to grow the ZnO nanoparticles as seed layer have been reported in our previous studies [22,23].

Several characterization methods have been developed to measure IFSS, including fiber push-in test [24], pull-out test [25], micro-bond test [26], and single-fiber fragmentation test (SFFT) [27]. It has been well accepted that SFFT is one of the most popular methods, as the specimen preparation and test operation are relatively simple, and the failure type can be directly visualized during experiments. Each SFFT specimen for the fragmentation test consists of one fiber encapsulated in the chosen polymer of the dogbone shape. Elongation of the SFFT specimen under tensile load results in fiber breakage. When the fiber embedded in the polymer gradually breaks into smaller fragments, the tensile stress at the fracture location reduces to zero, but the shear stress in the polymer remains constant. When the fragment length is too short to transfer enough stresses into fiber to cause further breakage, the number of fragments will become constant. Therefore, the maximum number of fragments after SFFT tests indicates the interfacial shear strength of fiber and polymer matrix in composites.

Numerical techniques have been employed to investigate hybrid composites [28]. Multiscale modeling was utilized to predict the mechanical properties of enhanced fiber-reinforced composites with aligned carbon nanotubes [29]. Kulkarni et al. reported that the grown nanoparticle on the fiber and embedded in the matrix creates an enhancement layer in which effective material properties can be evaluated by microscale homogenization analysis [30]. This method has been used to estimate the macroscale properties of hybrid composites, including mechanical behavior [31,32,33,34], piezoelectric properties [35], and thermal properties [36].

To the best of our knowledge, there is no integrated investigation of ZnO nanowires reinforced hybrid structural composites reported in the literature. In this paper, an experimental synthesis, characterization, and testing approach were combined with numerical analysis to fully understand the advantages of ZnO nanowire-enhanced hybrid structural composites. In particular, ZnO nanowires grown on carbon fibers were used to develop hybrid composites. Individual carbon fibers in the diameter of several micrometers were coated with ZnO nanowires. Nanoscale ZnO seeds were first deposited on a single carbon fiber using the ALD method; then, aligned 1D ZnO nanowires were synthesized using hydrothermal treatment. The synthesized ZnO nanowires on carbon fibers were characterized by field-emission scanning electron microscope (FESEM), energy-dispersive X-ray spectroscopy (EDX), X-ray diffraction (XRD), and thermogravimetric analysis (TGA). The influence of this selective, nanoscale reinforcement on the mechanical properties of fiber–matrix interfaces was investigated using the single-fiber fragmentation test. The ZnO nanowires with optimal diameter and length to diameter ratio resulted in improved fiber–matrix interfacial load transfer capability. The improvements in interfacial strength of the ZnO nanowire-reinforced composites can potentially increase the shear modulus and yield strength of polymer matrix composites. Moreover, multiscale modeling was carried out to investigate the improved IFSS of the hybrid SFC. Microscale homogenization was employed to extract the effective material properties of the ZnO nanowires grown on the fiber and embedded in an epoxy matrix (enhancement layer). The interface between fiber and the enhancement layer was simulated at mesoscale using the cohesive zone model (CZM). At the macroscale, the damage behavior of fiber was constructed utilizing user subroutine to define a material’s mechanical behavior (UMAT). Finite element analysis (FEA) of SFFT was performed in ABAQUS software, and the IFSS value was calculated.

## 2. Materials and Methods

### 2.1. Material

All chemicals were used as received. Diethylzinc (DEZn, Zn(C_2_H_5_)_2_) and deionized water (DI water) were received from Sigma-Aldrich (St. Louis, MO, USA), to grow the ZnO nanoparticles in the first step of the ALD method. Zinc nitrate hexahydrate (Zn(NO_3_)_2_ 6H_2_O) and hexamethylenetetramine (HMTA) were purchased from Sigma-Aldrich, to synthesize the ZnO nanowires in the second step of the hydrothermal method.

### 2.2. Synthesis of ZnO Nanoparticles

Single carbon fiber was stripped from carbon fabric, fixed on a Teflon frame by carbon tape. A customized ALD system was used to execute the growth of ZnO nanoparticles on a single carbon fiber at the temperature of 200 °C and a background pressure of 0.5 Torr, for a total of 300 growth cycles. The vapor ratio of DI water and DEZn was about 2, and ultrahigh purity N_2_ gas was purged after each dose for 20 s. These growth parameters were the optimized results reported in our previous publication [21].

### 2.3. Synthesis of ZnO Nanowires

Zn(NO_3_)_2_ and HMTA powder were dissolved in DI water to prepare the growth solutions. Each solution was heated to 90 °C and stirred at 800 rpm for 10 min on the hotplate and then combined together in a beaker. The single carbon fiber on the Teflon frame was immersed in the beaker covered with wrap. The beaker was stored in a water bath at 95 °C for 17 h. The solutions of concentration of Zn(NO_3_)_2_ and HMTA from 25 mmol/L, 50 mmol/L, and 100 mmol/L at a molar ratio of 1:1 were prepared to vary the morphologies of ZnO nanowires.

### 2.4. Characterization

The FESEM (Zeiss, Peabody, MA, USA) was used to investigate the morphologies of ZnO nanoparticles and nanowires. The software ImageJ was used to measure the length and analyze the statistical diameters of ZnO nanowires based on the FESEM images. EDX spectroscopy was employed to conduct the elemental analysis of hybrid carbon fiber. Rigaku Ultima IV diffractometer (Rigaku, Austin, TX, USA) was used to carry out the XRD measurement of the ZnO structure.

### 2.5. Single-Fiber Tensile Test

A single-fiber tensile test was carried out to measure the tensile strength of bare carbon fiber and carbon fiber with ZnO nanowires and to identify the effect of ZnO nanowires on carbon fiber. It is important to obtain this value to calculate the IFSS of the single-fiber fragmentation test. The major benefit of growing ZnO nanowires on carbon fiber is the preservation of the tensile strength of carbon fiber with a slight decrease. The gauge length of single bare carbon fiber and carbon fiber with ZnO nanowires is 25.4 mm, and 15 fibers of each were tested by DEBEN tensile stage (Deben, London, UK) with a 2N load cell applying a displacement rate of 0.1 mm/min.

### 2.6. Single-Fiber Fragmentation Test

SFFT is an essential technique to determine the interfacial properties between polymer matrix and carbon fiber [27]. A dog-bone specimen embedded with single carbon fiber inside was prepared to perform the tensile test under an optical microscope. Stress is transferred to the fiber in a shear direction when the specimen is in tensile status. Fiber fracture will occur when the applied external force exceeds its tensile strength. The new segmented fiber can continue to transfer load when the tensile force increase. The number of cracked carbon fiber will increase until it reaches saturation, which means the existed fiber segment cannot transfer the load to generate new fractures of carbon fiber. After saturation, the average IFSS can be calculated by following Equation (1).

(1) $$\tau =\frac{\delta_{f}d}{\frac{8}{3}\bar{l}}$$

where $\delta_{f}$ is the tensile strength of a single carbon fiber at the critical length,  d is the diameter of carbon fiber, and $\bar{l}$ is the average length of fragmentation.

Single carbon fiber was clipped by two small clamps and transferred to the middle of a silicone mold (Bluesil V-340/CA-45Mold Making Silicone Rubber, York County, VA, USA) before infiltrating the epoxy. Pretension was applied to the carbon fiber due to the shrinkage of epoxy during curing. Epon 862 and Epikure 9553 were used to prepare the polymer matrix due to its high tensile strain of over 9%, which is much larger than the tensile strain of carbon fiber. Moreover, this polymer keeps its transparency after curing, which helps the observation of counting cracks under an optical microscope. They were mixed at a weight ratio of 100:16.9, degassed in a vacuum tank for 3 min. The epoxy solution was infiltrated into the mold by a syringe to reduce the potential air bubble before curing. Then, the mold was left at room temperature for 16 h and put in the oven at 100 °C for 1 h and 160 °C for 1 h.

After curing, all specimens were polished by Struers with the following steps: SiC foil 320 sandpaper and MD GEKKO were used to remove extra materials and reduce the specimen thickness to 1.1 mm to meet the 200 N load cell capacity of the tensile stage. Then, the samples were polished on both sides by MD LARGO, with 9 micro-diamond sprays, MD MOL with 3 micro-sprays, and MD CHEM with silica suspension. Each of these three steps was polished for 3 min at 150 RPM under 30 N. Finally, specimens with smooth and transparent surfaces were obtained for later SFFT.

## 3. Experimental Results and Discussion

Multiple synthesis parameters including ALD background pressure, ALD temperature, ALD growth time, number of ALD cycles, the concentration of zinc nitrate hydrate and HMTA, and hydrothermal treatment time and temperature have critical effects on the morphologies of ZnO nanowires. Based on our previous report, lower temperature, higher growth time, and higher concentration of zinc nitrate hydrate and HMTA can result in an increase in the average diameter of ZnO nanowires. Undesired ZnO nanorods or nanoflakes occur when the ZnO diameter is large, which indicates the closely grown ZnO nanowires can prevent the infiltration of liquid epoxy. Considering multiple parameters can result in a large group of ZnO nanostructures in different morphologies, in this study, we employed constant parameters of the ALD process, and only varied the concentration of Zn(NO_3_)_2_ and HMTA, with values of 25 mmol/L, 50 mmol/L, and 100 mmol/L, at 95 °C for 17 h, to investigate its effects.

**Figure 1:**
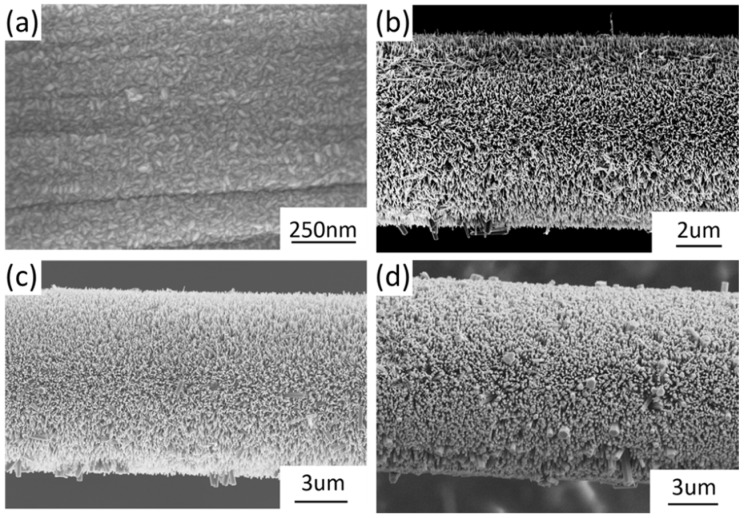
FESEM images of ZnO nanostructure on single carbon fiber: (**a**) ZnO nanoparticles via ALD, (**b**) ZnO nanowires via 25 mmol/L, (**c**) Zn nanowires via 50 mmol/L, and (**d**) ZnO nanowires via 100 mmol/L.

The shape of ZnO nanoparticles as seed layer was rice-like with random orientation, as illustrated in Figure 1a. The length of ZnO nanoparticles ranged from 15 nm to 30 nm, and the width ranged from 7nm to 15 nm. ZnO nanowires vertically grew on the surface of a single carbon fiber with relatively uniform length and diameter, but some large nanowires were observed in a random distribution. When the reagent concentration increased, the morphologies of ZnO nanowires changed from needle-like tips (Figure 1b) to hexagonal columns (Figure 1d), and the length of ZnO nanowires varied greatly. The length of ZnO nanowires was defined as the half value of the diameter of carbon fiber, with ZnO nanowires subtracting the diameter of bare carbon fiber. The diameter of bare carbon fiber was 6.972 µm, so the average length of ZnO nanowires under different growth conditions was 0.811 µm, 1.853 µm, and 2.490 µm, respectively. It is noted that the sizes of ZnO nanowire increased as the hydrothermal reagent concentration increased. Since the same process temperature and time were used, the reagent concentration was the dominant parameter that controlled the tomography of synthesized ZnO nanowires. Our previous publications have reported the effects of hydrothermal process time and temperature [23]. All SEM images were taken using a high-resolution Zeiss Neon SEM system with an accelerating voltage of 5 kV.

**Figure 2:**
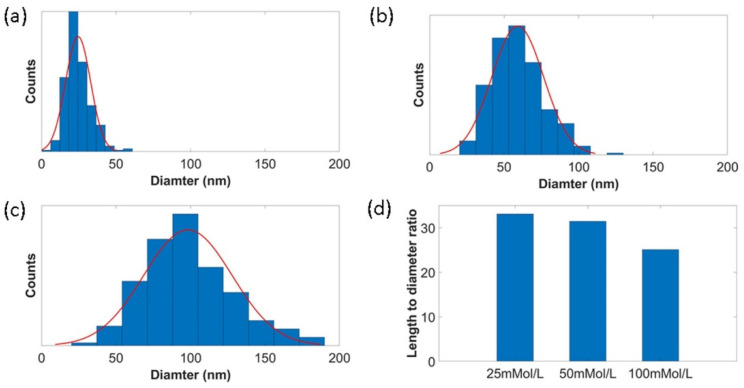
Diameter distribution of ZnO nanowires under different concentrations: (**a**) 25 mmol/L, (**b**) 50 mmol/L, (**c**) 100 mmol/L, and (**d**) length-to-diameter ratio of each concentration.

Much higher magnification (30 k to 60 k) of FESEM was taken, and ImageJ software was used to measure the diameter of ZnO nanowires from corresponding images. The diameter distributions of ZnO nanowires under different growth conditions are manifested in Figure 2a–c. By increasing the reagent concentration, the average diameters of ZnO nanowires increased. The average diameter of ZnO nanowires was 24 nm, 58 nm, and 99 nm when the used reagent concentration was 25 mmol/L, 50 mmol/L, and 100 mmol/L. The largest ZnO diameter observed was almost close to 200 nm, which is shown in Figure 1d. We believe that the dispersity of diameter was related to the inhomogeneous rice-like ZnO nanoparticles synthesized in the ALD process. Figure 2d shows the average length to diameter ratio of ZnO nanowires under different concentrations. The length to diameter ratios of ZnO nanowires were 33, 31, and 25 when the reagent concentrations were 25 mmol/L, 50 mmol/L, and 100 mmol/L, respectively. It can be seen that the increased growth concentration resulted in a smaller length-to-diameter ratio of ZnO nanowires.

**Figure 3:**
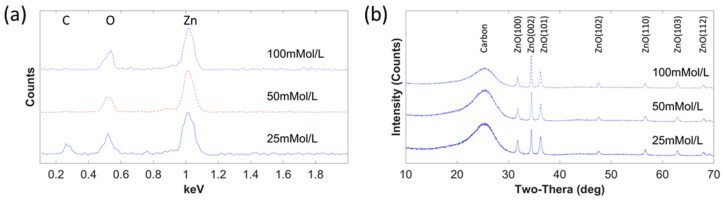
(**a**) EDX spectrum of ZnO nanowires on single carbon fiber; (**b**) XRD diffraction of ZnO nanowires with different morphologies.

EDX analysis was performed to detect the material composition of ZnO nanowires on a single carbon fiber. Figure 3a illustrates the EDX spectrum of ZnO nanowires on carbon fiber under different growth concentrations. It is observed that zinc and oxygen elements were all detected for three groups, but carbon was almost missing when the reagent concentration was 100 mmol/L due to the high volume of ZnO with the largest length and diameter on carbon fiber. The peak height of zinc increased, compared with the peak height of oxygen when the growth concentrations increased, so did the relationship between oxygen and carbon. The EDX results confirmed that the nanowires on carbon fiber were formed by ZnO.

The crystal structure of ZnO nanowires was investigated by XRD, as shown in Figure 3b. The record range was between 10° and 70°, with a speed of 2 degrees/min at a scanning step of 0.02°. For the ZnO nanowires grown in the concentration of 100 mmol/L, 2θ values of 31.8°, 34.48°, 36.3°,47.6°, 56.62°, 62.88°, and 67.98° were found for diffraction peaks, which were identified for (100), (002), (101), (102), (110), (103), and (112) of the ZnO planes, respectively. There was a slight difference in 2θ peak for each concentration, which is difficult to discern in the figure. JCPDS card number 80-0075 was used to identify all the XRD diffraction patterns of ZnO nanowires, which showed that ZnO nanowires were wurtzite crystal structures. Of all the diffraction patterns, planes of (100), (002), and (101) dominated the main crystal structures. With an increase in reagent concentration, the intensity of all ZnO peaks increased, compared with the intensity of carbon. Within the three dominating peaks, peak (002) improved strongly, and peaks (100) and (101) became weaker, compared with peak (002). The intense peak of the (002) plane shows that the preferred growth orientation of ZnO nanowires was along the c-axis direction.

**Figure 4:**
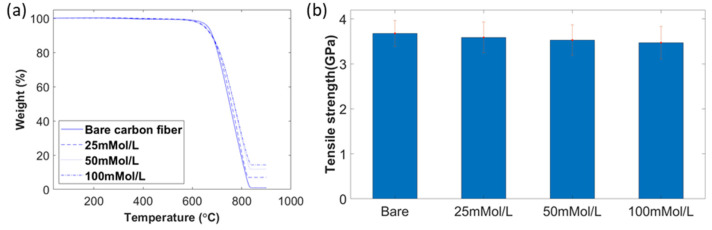
(**a**) TGA of bare carbon fiber and carbon fiber with ZnO nanowires; (**b**) tensile strength of bare carbon fiber and carbon fiber with ZnO nanowires.

In order to identify ZnO nanowire weight concentration, a TGA analysis (Texas Instruments, Austin, TX, USA) was performed. Due to the high decomposition temperature of ZnO (1974 °C), compared with that of carbon fiber (less than 700 °C in air), all carbon fibers were burnt off before the decomposition of any ZnO nanowires [37]. The temperature range of the TGA test was from 35 °C to 900 °C with a ramp of 10 °C/min. As shown in Figure 4a, the starting decomposition temperature of all carbon fiber with ZnO nanowires was around 600 °C, which was lower than the deposition temperature of bare carbon fiber at 620°C. After all carbon fiber was burned off, the residual weight percentage of each was 0.92%, 7.01%, 11.82%, and 14.33%, which indicated that the growth of higher reagent concentration resulted in a higher weight percentage of ZnO. There was no weight loss before the temperature rose to 300 °C, which meant no moisture was inside. Less than 1% of weight loss was found when the temperature ranges from 300 °C to 550 °C, which was caused by the burn-off of the sizing on carbon fibers.

The tensile strength of single carbon fiber under different concentrations is demonstrated in Figure 4b. The tensile strengths of bare carbon fiber and carbon fiber under the growth concentrations of 25 mmol/L, 50 mmol/L and 100 mmol/L were 3.676 ± 0.286 GPa, 3.588 ± 0.343 GPa, 3.529 ± 0.337 GPa, and 3.47 ± 0.362 GPa, respectively. Since the highest ALD process temperature was only 200 °C, there was no significant degradation of carbon fiber with ZnO nanowires under different growth concentrations. The slight decrease in tensile strength was caused by the fracture of ZnO nanowires.

**Figure 5:**
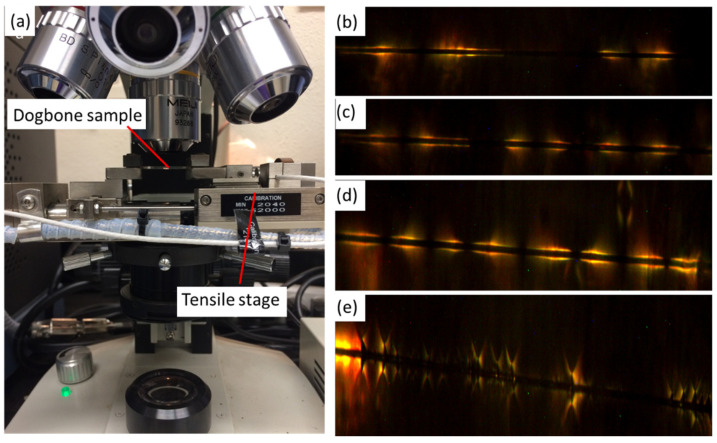
(**a**) Setting up of single-fiber fragmentation test, crack pattern of bare carbon fiber and carbon fiber with ZnO nanowires under different growth concentrations: (**b**) bare carbon fiber, (**c**) 25 mmol/L, (**d**) 50 mmol/L, and (**e**) 100 mmol/L.

The setting up of SFFT is demonstrated in Figure 5a. The specimen was fixed on the tensile stage with a 200 N load cell, and the gauge length of the dog-bone sample was 15 mm applied with the strain rate of 1 mm/min. The quantity of cracked fiber was counted by the feature of birefringence under the optical scope at 10× magnification through polarized light. The quantity of fragmentation was recorded after every 0.1 mm displacement was applied until saturation was observed multiple times. In Figure 5b–e, debonding of adhesive failure was seen. Under a similar magnification, only two cracks were observed from bare carbon fiber, but more fractions were observed for the carbon fiber with ZnO nanowires.

**Figure 6:**
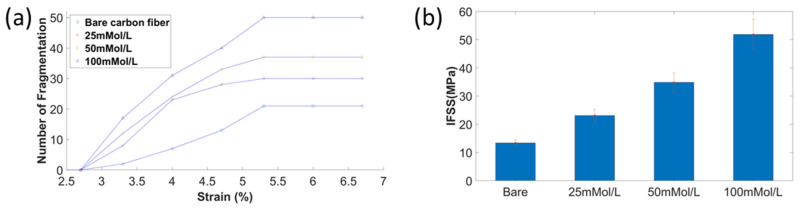
(**a**) number of fragmentations of carbon fiber with and without ZnO nanowires; (**b**) IFSS of carbon fiber without and with ZnO nanowires under different growth concentrations.

Hybrid composites with increased ZnO nanowires diameters showed enhanced interfacial properties, as the number of saturated fragmentations increased significantly, as shown in Figure 6a. The fragmentation number of bare carbon fiber in saturation was 21, and the increased number of fragmentations was 50 for the carbon fiber with ZnO nanowires under the growing concentration of 100 mmol/L. The first cracks of all specimens occurred when the applied tensile strain was at 3.33%, which was much higher than the general tensile strain (1.7–2.2%). This was due to the applied pretension load during the specimen curing was not enough to keep the fiber aligned. The saturation strain for all specimens was 5.33%. After reaching saturation, no additional fragmentations of carbon fiber were found when continuing to increase the tensile strain up to 6.67%.

It is noted that the increased fragmentations led to the reduced average length of fragmentations $\bar{l}$, and the increased length of ZnO nanowires resulted in a larger diameter of carbon fiber d. The tensile stress of carbon fiber changed little, based on the calculation equation of interfacial shear stress considering all these factors; therefore, IFSS was greatly improved, changing from 13.4 ± 1.1 MPa to 51.8 ± 5.4 MPa, as demonstrated in Figure 6b. The increased ZnO nanowire diameters and length caused increased volume for load transfer and a reduced contact area between carbon fiber and epoxy. IFSS increased when the diameter and length of ZnO nanowires increased, mainly caused by the increased interfacial area and mechanical interlocking between carbon fiber and epoxy. ZnO nanowires worked as an interphase between carbon fiber and polymer matrix to improve the bonding.

## 4. Multiscale Modeling of Hybrid SFFT

**Figure 7:**
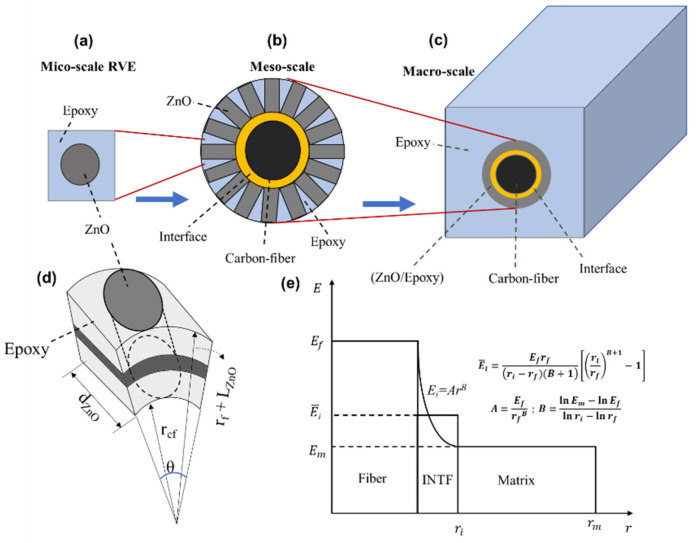
Schematic of (**a**,**d**) RVE used for homogenization of ZnO/epoxy layer; (**b**) radially aligned ZnO on the fiber and embedded in epoxy; (**c**) different phases containing fiber, interface, coating layer, and epoxy; (**e**) distribution of interface stiffness.

A 3D multiscale modeling approach was developed to simulate the SFFT of the enhanced carbon fiber with radially aligned ZnO nanowires. Considering the diverse length scale of the materials used in the hybrid single-fiber composites, a numerical analysis was performed at different length scale zones, as shown in Figure 7a–c. The ZnO nanowires aligned on the fiber surface and embedded in the epoxy matrix were modeled as an enhancement coating layer. The microscale homogenization method was utilized to extract the effective material properties of the ZnO–epoxy layer. Hence, a rectangular representative volume element (RVE) was modeled containing a single ZnO nanowire in the epoxy domain, and the result is used for the next step (Figure 7a). Based on the compact radial alignment of the nanowires in the model shown in Figure 7b,d, the maximum volume fraction of the ZnO nanowire in the coating layer can be derived from Equation (2). More information about homogenization analysis and extracting the material properties of the coating layer can be found in [17,38].

(2) $$\nu_{\mathrm{ZnO}}=\frac{n_{\mathrm{ZnO}}\left(V_{one\;\mathrm{ZnO}}\right)}{V_{Coating\;layer}}=\frac{\frac{\pi d_{cf}}{d_{\mathrm{ZnO}}}\left(\pi \frac{d_{\mathrm{ZnO}}^{2}}{4}L_{\mathrm{ZnO}}\right)}{\pi d_{\mathrm{ZnO}}\left[{\left(R_{cf}+L_{\mathrm{ZnO}}\right)}^{2}-R_{cf}^{2}\right]}$$

The interface between the fiber and the enhanced coating layer was simulated via the cohesive zone model. The bilinear traction–separation law was utilized in which the cohesive stiffness (*K*) increases by applying the load up to the separation displacement (*δ_c_*). Then, the damage initiates in the interface, and the cohesive stiffness degrades linearly up to the final displacement (*δ_t_*) as described in Equation (3). The degradation is controlled by the fracture energy, which is the area under the stress–displacement curve. The maximum nominal stress is chosen as a damage criterion according to which the damage initiates when the maximum stress exceeds the interface strength. The values of the fracture energy and maximum stress were assumed as *σ_max_* = 50 MPa and *G_Ic_ =* 100 J/m^2^ in this study [39,40]. Moreover, the power-law variation model was implemented for defining the interface elastic modulus based on the modulus of the fiber and the surrounding area, and the average value through the thickness ($\bar{E_{i}}$) was considered as the stiffness, as shown in Figure 7e.

(3) $$\sigma_{Int.}=\left(1-D\right)K\delta ,\;\;D=max\left\{\begin{array}{c} 0\\ \frac{\left(\delta -\delta_{c}\right)\delta_{t}}{\left(\delta_{t}-\delta_{c}\right)\delta_{t}}\\ 1 \end{array}\begin{array}{c} \;\;\;\;\;\;\;\;\;\delta \leq \delta_{c}\;\;\\ \;\;\;\;\;\;\;\;\;\delta_{c}\leq \delta \leq \delta_{t}\\ \delta \geq \delta_{t} \end{array}\right\}$$

A 3D hybrid composite with a length of 0.5 mm and a width of 0.28 mm was considered. A quarter of the sample was simulated in the ABAQUS FEA software based on two symmetric planes. Two models were investigated—a bare fiber and an enhanced fiber with radially aligned ZnO nanowires with a diameter of 24 nm and length of 0.81 μm (similar to the case of 25 mmol/L). According to the model provided by Chen et al. [41], Young’s modulus and Poisson’s ratio of ZnO are 205 GPa and 0.33, respectively. A thin layer of interface with a thickness of 0.01 μm was implemented to simulate the interface between the fiber and the coating layer. A 3D cohesive element with 8 nodes (COH3D8) was utilized for the interface, while an 8-node linear brick element (C3D8S) was used for the fiber, ZnO–epoxy coating layer, and the matrix. Based on Equation (2), the maximum volume fraction of ZnO in the coating layer used for the homogenization analysis is 70.28%. In order to simulate the SFFT, the maximum stress failure criterion was implemented to simulate fiber failure using the ABAQUS UMAT user subroutine [42], programmed in FORTRAN. According to this theory, and assuming the carbon fiber as transversely isotropic material, the damage occurs in the fiber when (*σ*_1_ ≥ σ_t_* or *σ*_1_ ≤ σ_c_*) or (*τ*_12_ ≥ τ*), where σ_t_*, σ_c_*, and τ* are the tensile strength, compressive strength, and shear strengths of the fiber, respectively. If the failure criterion satisfies, the related elements cannot carry the load, and the fiber breaks into two segments. The constitutive failure continues until reaching the saturation level, and the analysis then stops. The material properties of the fiber, ZnO, and epoxy used for the FEA are defined in Table 1.

## 5. Numerical Results

**Figure 8:**
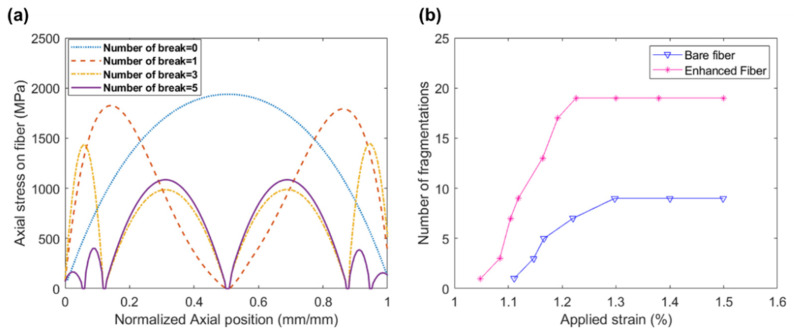
(**a**) Axial stress in the fiber at different fragmentation steps; (**b**) fragmentation density for bare and enhanced fiber.

The homogenization analysis was performed first, and the effective material properties of the ZnO–epoxy coating layer were extracted and used for the SFFT simulation. By increasing the axial load, the fiber was broken into different segments based on the damage criterion. The axial stress over the fiber at different applied strains calculated for the enhanced fiber is shown in Figure 8a. It is obvious that breakage occurred in the middle of the fiber. After breakage, each segment behaved as an independent fiber, and the next fracture occurred in the middle of each part.

The constitutive fiber failure continued until the saturation level. The fiber fragmentation density for the enhanced fiber with ZnO coating is compared with the bare fiber in Figure 8b. According to this figure, the number of fragmentations in the enhanced fiber (n = 19) is almost two times the bare fiber (n = 9). Based on Equation (1), the interfacial shear strength of the bare fiber is increased by 99% by adding the radially aligned ZnO nanowires. By comparing the numerical results (Figure 8b) with the experimental analysis (Figure 6), it can be seen that this improvement extracted from FEA is very close to the experimental results for the hybrid fiber with ZnO nanowires (d = 24 nm, L = 0.81 μm).

## 6. Conclusions

In this study, we investigated a novel approach to synthesizing ZnO nanowires using integrated ALD and hydrothermal treatment as enhanced interphase between carbon fiber and polymer matrix for structural composite applications. The diameters of ZnO nanowires in the range of 24.491 nm to 99.303 nm and their lengths in the range of 0.811 um to 2.49 µm were controlled by adjusting the growing concentration of Zn(NO_3_)_2_ and HMTA. No carbon fiber degradation was observed on developed hybrid carbon fiber due to low nanowire synthesis temperatures. IFSS was greatly improved, by 286%, with the incorporation of ZnO nanowires. This result proved that growing ZnO nanowires can be a practical approach to the enhancement of the bonding between carbon fiber and polymer matrix. Additionally, a multiscale damage analysis was conducted to better understand the interfacial properties of the developed hybrid composites. The homogenization method was used to evaluate the effective material properties of the enhanced coating layer (ZnO nanowires grown on the fiber and embedded in epoxy). The cohesive zone method was employed to simulate the interface between the fiber and enhancement layer. The maximum stress theory was utilized to simulate the fiber failure using the UMAT user subroutine. The SFFT analysis of 3D enhanced SFC with the ZnO diameter of 24 nm and the length of 0.81 μm (such as the case of 25 mmol/L in experiments) was simulated in ABAQUS. About 99% improvement in IFSS was observed by comparing the results of the bare and the enhanced SFC, which was very close to the experimental results. Both experimental and numerical analyses demonstrated the improvement of load transferring capability by growing ZnO nanowires on carbon fiber for structural composite applications.

## Figures and Tables

**Table 1:** Material properties of fiber, ZnO, and epoxy used for the FEA.

Parameter	*E_fiber_* (GPa)	*ν_fiber_*	σ_t_^*^_fiber_ (MPa)	σ_c_^*^_fiber_ (MPa)	τ^*^_fiber_ (MPa)	*E_Epoxy_* (GPa)	*ν_fiber_*	*E_ZnO_* (GPa)	*ν_ZnO_*	*E_fiber_* (GPa)
Value	232	0.2	2000	1450	1200	3.5	0.3	205	0.33	232

## Data Availability

Not applicable.
